# Microfluidic Biosensor Based on Molybdenum Disulfide (MoS_2_) Modified Thin-Core Microfiber for Immune Detection of *Toxoplasma gondii*

**DOI:** 10.3390/s23115218

**Published:** 2023-05-31

**Authors:** Huiji Chen, Binbin Luo, Shengxi Wu, Shenghui Shi, Qin Dai, Zehua Peng, Mingfu Zhao

**Affiliations:** 1 Chongqing Key Laboratory of Optical Fiber Sensor and Photoelectric Detection, Chongqing University of Technology, Chongqing 400054, China; chenhuiji@stu.cqut.edu.cn (H.C.); shshill@cqut.edu.cn (S.S.); zehua.peng@stu.cqut.edu.cn (Z.P.); zmf@cqut.edu.cn (M.Z.); 2 School of Pharmacy and Bioengineering, Chongqing University of Technology, Chongqing 400054, China; 416047053@stu.cqut.edu.cn

**Keywords:** thin-core microfiber, microfluidic chip, molybdenum disulfide, optical fiber biosensor, *toxoplasma gondii*

## Abstract

**Simple Summary:**

A microfluidic biosensor based on a thin-core microfiber coated with molybdenum disulfide for immune detection of *Toxoplasma gondii* was illustrated. The detection range, limit of detection, detection sensitivity, specificity, and clinical properties of the proposed biosensor were explored.

**Abstract:**

*Toxoplasma gondii* (*T. gondii*) is a zoonotic parasite that is widely distributed and seriously endangers public health and human health. Therefore, accurate and effective detection of *T. gondii* is crucial. This study proposes a microfluidic biosensor using a thin-core microfiber (TCMF) coated with molybdenum disulfide (MoS_2_) for immune detection of *T. gondii*. The single-mode fiber was fused with the thin-core fiber, and the TCMF was obtained by arc discharging and flame heating. In order to avoid interference and protect the sensing structure, the TCMF was encapsulated in the microfluidic chip. MoS_2_ and *T. gondii* antigen were modified on the surface of TCMF for the immune detection of *T. gondii*. Experimental results showed that the detection range of the proposed biosensor for *T. gondii* monoclonal antibody solutions was 1 pg/mL to 10 ng/mL with sensitivity of 3.358 nm/log(mg/mL); the detection of limit was calculated to be 87 fg/mL through the Langmuir model; the dissociation constant and the affinity constant were calculated to be about 5.79 × 10^−13^ M and 1.727 × 10^14^ M^−1^, respectively. The specificity and clinical characteristics of the biosensor was explored. The rabies virus, pseudorabies virus, and *T. gondii* serum were used to confirm the excellent specificity and clinical characteristics of the biosensor, indicating that the proposed biosensor has great application potential in the biomedical field.

## 1. Introduction

*Toxoplasma gondii* (*T. gondii*) is a relatively common intracellular parasite among human parasitic infection species that generally exists in warm-blooded animals and belongs to the opportunistic pathogenic protozoa of zoonosis. It not only causes huge economic damage to agricultural production but also seriously endangers food safety and human health. It is estimated that more than one third of humans have long-term latent infection with *T. gondii* [1,2]. It is worth noting that most patients infected with *T. gondii* are asymptomatic, but in immunocompromised patients, primary or recurrent infections can lead to life-threatening encephalitis. Moreover, the abortion rate of pregnant women infected with *T. gondii* is high, and it can also be spread to the fetus through the placenta during pregnancy. In order to prevent infection of *T. gondii* in a timely and accurate manner and to protect the safety of life and property, it is urgent to study the detection method of *T. gondii*. The detection of *T. gondii* through a microscope is less sensitive and requires more expertise, while the method of inoculation is time-consuming (usually taking several weeks) and is expensive to test [3,4]. Enzyme-linked immunosorbent assay (ELISA) is convenient, repeatable, and easy to automate. However, the development of ELISA is labor-intensive when it comes to assessing sensitivity and specificity [3]. Polymerase chain reaction (PCR) is based on nucleic acid amplification and is very attractive because of its accuracy, sensitivity, and reliability. However, the detection process is relatively cumbersome and it amplifies the DNA of live and dead parasites, leading to overestimation of the number of parasites in the blood [5,6,7]. Therefore, it is of great significance to explore an accurate, rapid, and effective method for the detection of *T. gondii* with high specificity, simple operation, and high sensitivity. 

Optical fiber biosensors can detect biological molecules such as enzymes, antigens/antibodies, and DNA through the optical transduction mechanism; this has the advantages of compact structure, high sensitivity, and label-free detection. It is a good platform for detecting biological substances [8,9,10]. However, with the increasing demand for high sensitivity, more and more special optical fibers are applied in optical fiber biosensors to improve the sensing performance and achieve ultra-high sensitivity [11,12,13]. Thin-core fiber (TCF) is a special single-mode fiber (SMF) with a smaller core diameter or cladding diameter than the standard SMF. When the TCF and SMF are fused together, the high-order cladding modes are easier to be excited at the interface because of the mismatch of the core diameter, enhancing the interaction between optical signal and surroundings. In particular, the thin-core microfiber (TCMF) prepared by TCF has the characteristics of simple fabrication, compact structure, strong evanescent field effect, and light volume. It is an excellent carrier for the detection of *T. gondii* and suitable for the field of biosensing. 

In recent years, two-dimensional materials such as graphene oxide (GO) and molybdenum disulfide (MoS_2_) have been widely used in the field of biosensing due to their high conductivity, good biocompatibility, large surface-to-volume ratio, and non-toxicity; as a result of these properties, they can detect micro-biomolecules at low concentrations and improve the sensitivity and biocompatibility of sensing platform [14,15]. In 2020, Fang et al. [16] proposed a microfiber sensor coated with GO encapsulated in a microfluidic chip made by polydimethylsiloxane (PDMS) to detect hemoglobin. Moreover, MoS_2_ has the advantages of high carrier mobility and the existence of free sulfur groups in the hydrophobic interaction, which provides more bond sites for immobilization of antibodies and improves sensitivity. In 2019, Kaushik et al. [17] dipped a gold coated multimode optical fiber etched with hydrofluoric acid into an MoS_2_ nanosheets solution to obtain the MoS_2_/Au/optical fiber sensor for the detection of bovine serum albumin. In 2021, Li et al. [18] demonstrated a biosensor based on multicore fiber and multimode fiber functionalized by graphene oxide, gold nanoparticles, MoS_2_, and creatininase enzyme to detect creatinine. 

This work proposes and demonstrates a biosensor based on thin-core microfiber (TCMF) encapsulated in self-made microfluidic chip. Although the self-made microfluidic chip may have limitations such as lack of standardization and poor universality, it protects the sensor from external interference and provides a stable sensing environment. In the experiment, the TCMF obtained by arc discharging and flame heating was first modified with MoS_2_ followed by its bio-functionalization with *T. gondii* antigens (*T. gondii* Ag). The abrupt taper excited the fundamental mode and high-order cladding modes to form interference. The combination of antibodies and antigens caused the effective refractive index (RI) of different modes to change, resulting in wavelength shift. The detection range, limit of detection, detection sensitivity, specificity, and clinical properties of the biosensor were explored through the immunodetection of *T. gondii* monoclonal antibodies (*T. gondii* MAb). Based on the advantages of simple structure and convenient fabrication, the proposed sensor shows promising applications in biosensing fields. 

## 2. Methodology and Experiments

### 2.1. Materials and Instruments

Thin-core fiber (TCF, CS1015-B, core diameter: 3.8 µm, cladding diameter: 125 µm) was purchased from Wuhan Changfei Optical Fiber and Cable Co., Ltd. in China. The core and cladding diameters of single-mode fiber (SMF, Corning) were 8.1 µm and 125 µm, respectively. The chemical reagents used in the experiment were analytical reagents, and all working solutions were prepared with sterile deionized water. Sodium hydroxide (NaOH) was purchased from Chongqing Chuandong Chemical Co., Ltd. in China. (3-mercaptopropyl). Trimethoxysilane (MPTMS) was purchased from Shanghai Aladdin Biochemical Technology Co., Ltd. in China. Nitric acid (HNO_3_) and sodium chloride (NaCl, the purity is 99%) were purchased from Sigma-Aldrich, China. Lamina molybdenum disulfide (MoS_2_) dispersion was purchased from Nanjing XFNANO Materials Technology Co., Ltd. in China. Phosphate buffered solution (PBS, 0.01 M, PH7.4) was purchased from Wuhan Boster Biological Technology Co., Ltd. in China. The home-made skimmed milk powder sealing fluid (SMPSF) was prepared from skimmed milk powder and PBS. The pseudorabies virus, rabies virus, and *T. gondii* MAb solutions, *T. gondii* Ag solution, and *T. gondii* serum were provided by the School of Pharmacy and Bioengineering, Chongqing University of Technology, Chongqing, China. 

The transmission spectrum characteristics of the proposed biosensor were investigated using an optical spectrum analyzer (OSA, YOKOGAWA AQ6370D, 600–1700 nm, Japan) and a broadband source (BBS, CONQUER ASE, SuZhou, China). A fusion splicer machine (TYPE-81C, Fujikura, Japan) was used to splice optical fibers. A high precision fiber optic cleaver (CT-38, Fujikura, Japan) was used for cleaving optical fibers. The fusion device (KL-300 T, JILONG, China) and the optical fiber pulling machine (OB-612) were used for arc discharging and flame heating. High resolution electron microscopy (SMZ800, Nikon, Japan) was used to observe the tapered structure of thin-core microfiber. Field emission scanning electron microscopy (FESEM, ZEISS Sigma HD^TM^, Jena, Germany) was used to characterize the surface of the MoS_2_-coated thin-core microfiber, and FESEM-EDS was used to determine the chemical element composition of the surface substances in the microscopic region. The RI was measured using an Abel refractometer (ATAGO, NAR-1 T SOLID, 1.3000–1.7000). 

### 2.2. Working Principle of the Thin-Core Microfiber

**Figure 1:**
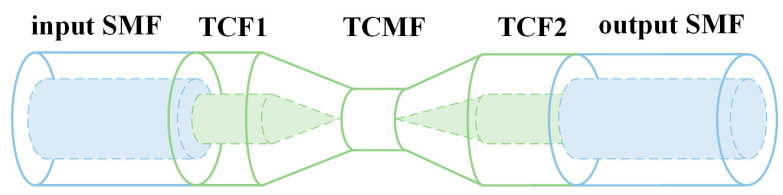
Schematic diagram of the TCMF sensor.

Figure 1 shows the schematic diagram of the TCMF sensor. A light signal is transmitted into the input SMF in the modality of fundamental mode when TCF is fused with the SMF. The TCF1 acts as a beam expander for incident light. Due to the mismatch of core diameters between TCF and SMF, high-order cladding modes are excited at the interface. The diameters of core and cladding of TCF are reduced simultaneously with the process of arc discharging and flame stretching; meanwhile, light energy in the core gradually leaks into the cladding and is guided by the cladding-to-air interface. Finally, the fundamental mode and excited high-order cladding modes are recoupled into the output SMF. The interference spectrum caused by different optical path differences between fundamental mode and different high-order modes was observed by OSA. 

Light intensity of the interference spectrum can be expressed as [19]

(1) $$I=I_{1}+I_{2}+2\sqrt{I_{1}I_{2}}\cos\frac{2\pi \Delta n_{eff}L}{\lambda }$$

where *I*_1_ and *I*_2_ are light intensities of the fundamental mode and excited cladding modes respectively, λ is the wavelength, *L* is the interference length, and $\Delta n_{eff}$ is the difference between the effective RI of the fundamental mode and cladding mode. 

The wavelength dip $\lambda_{dip}$ can be expressed as

(2) $$\lambda_{dip}=\frac{2\Delta n_{eff}L}{2m+1}$$

where *m* is an integer. The effective RI of high-order cladding mode changes with the external environment parameters; thus, $\Delta n_{eff}$ varies with the environmental RI, resulting in the wavelength shift of transmission spectrum. The RI sensitivity of wavelength dip can be calculated through the following equation by transforming Equation (2) [20]

(3) $$S=\frac{d\lambda_{dip}}{dn_{sur}}=\frac{\lambda_{dip}}{G}\cdot \frac{\partial \Delta n_{eff}}{\partial n_{sur}}$$

where $n_{sur}$ is the RI of the external environment, *G* is the group effective RI difference, which is expressed as

(4) $$G=\Delta n_{eff}-\lambda_{dip}\cdot \frac{\partial \Delta n_{eff}}{\partial \lambda_{dip}}$$

Moreover, the wavelength shift is not only affected by the change of environmental RI, but also by the change of cladding radius caused by the adhesion of biological substances on the surface of TCMF in the immune detection of *T. gondii*. Therefore, the wavelength shift can be expressed as [21]

(5) $$d\lambda_{dip}=\frac{\lambda_{dip}}{G}\cdot \frac{\partial \Delta n_{eff}}{\partial n_{sur}}dn_{sur}+\frac{\lambda_{dip}}{G}\cdot \frac{\partial \Delta n_{eff}}{\partial r}dr$$

### 2.3. Fabrication and Encapsulation of the Sensor

**Figure 2:**
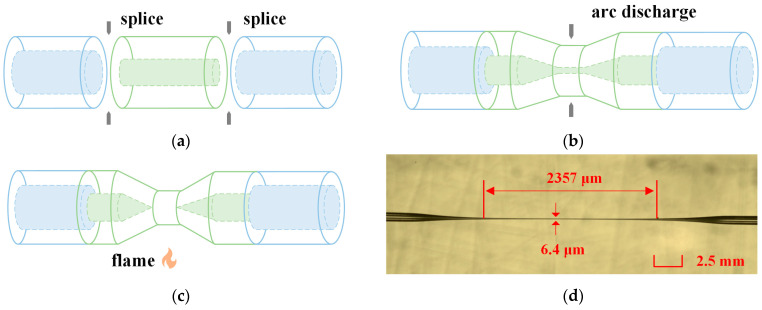
(**a**–**c**) Fabrication processes of the TCMF. (**d**) Micrograph of the TCMF.

**Figure 3:**
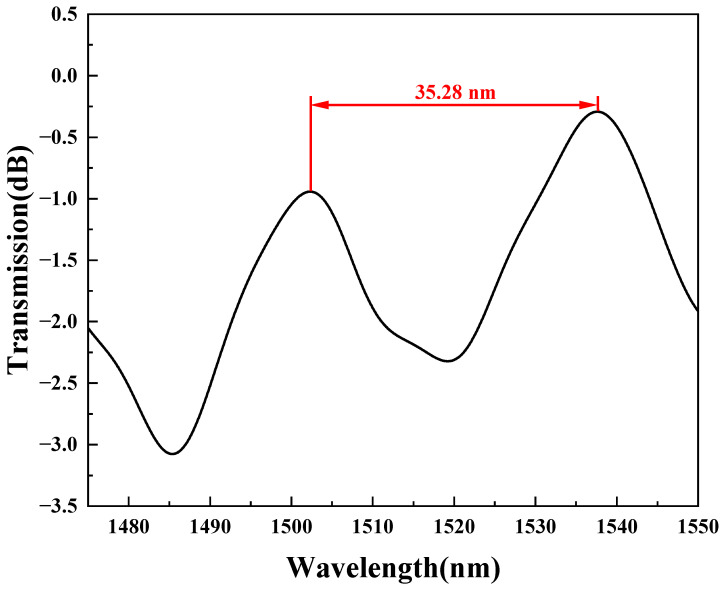
Transmission spectrum in air of the TCMF.

Figure 2a–c show the fabrication processes of the TCMF, which is the same as the previous work [22]. First, the TCF was spliced between two SMFs with a fusion splicer machine. It was easy for the TCF and SMF to splice without any processing because of the same cladding diameters. Next, a nonadiabatic taper based on the TCF was obtained by arc discharging with a fusion device. Finally, the nonadiabatic taper was tapered through hydrogen flame heating with an optical fiber pulling machine to obtain TCMF. The interference spectrum was monitored in real time during the whole process with the OSA and BBS. A micrograph of the TCMF is shown in Figure 2d. The waist diameter and waist length of TCMF in this work were chosen to be about 6.4 μm and 2357 μm, respectively. Figure 3 shows the transmission spectrum of the TCMF in air, and the free spectral range (FSR) is 35.28 nm. 

**Figure 4:**
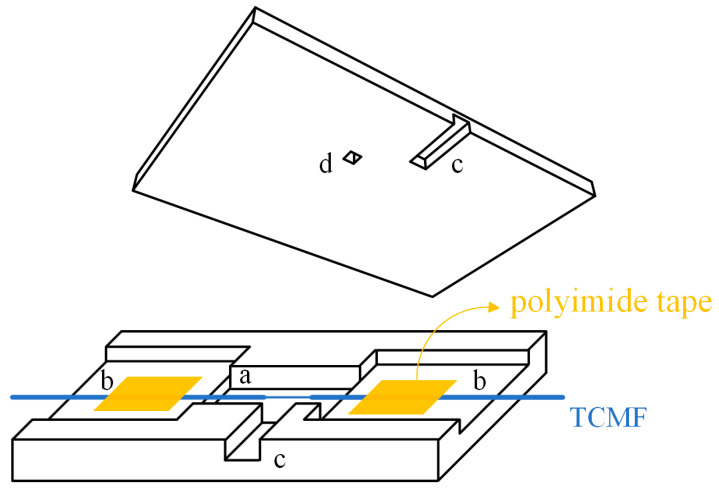
Structure design diagram of microfluidic chip.

It is worth noting that when most of the reported biosensors are placed in air or open experimental systems, the performance of these sensors will be affected by the surrounding environment, resulting in immeasurable experimental errors. Meanwhile, considering that the TCMF with an excessively small waist diameter is fragile, it is essential to encapsulate the proposed sensor. Here, a home-made microfluidic chip was fabricated to encapsulate the sensor to create an undisturbed sensing environment. The microfluidic chip was prepared by the PDMS with good biocompatibility, and its structural design is shown in Figure 4. The microfluidic chip is 60 mm in length, 40 mm in width, and 7 mm in height. The heights of bottom plate and lid are 5 mm and 2 mm, respectively. The waist area of TCMF was placed in the cuboid groove with a length of 20 mm, a width of 5 mm, and a height of 3 mm (Figure 4a) of bottom plate of the microfluidic chip, and both sides were pasted in the adhesion area (Figure 4b) by polyimide tape, as shown in Figure 4. The bottom plate and lid can be automatically bonded together without any adhesive because of the viscidity of the PDMS. The experimental reagents were injected into the cuboid groove from the liquid inlet (Figure 4c) by the syringe and the reagents were sucked out from the liquid outlet (Figure 4d) of the lid by the syringe after the operation was completed. The liquid inlet is 17.5 mm in length, 2 mm in width, and 4 mm in height, while the liquid outlet is a circular hole with a diameter of 1.5 mm. The bottom plate and lid together form a confined space, where the sensor can be encapsulated to avoid the disturbance of the surroundings and reduce the experimental errors. The self-made microfluidic chip accurately controls the dosage of experimental reagents and improves the robustness and stability of the proposed sensing structure. 

### 2.4. Surface Functionalization and Characterization of TCMF

**Figure 5:**
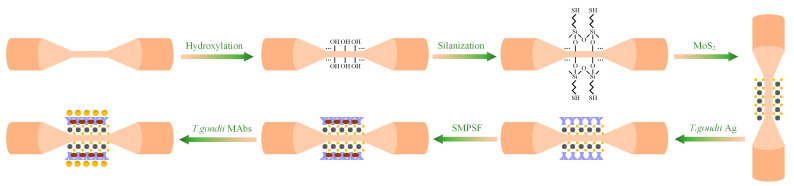
Schematic of modification process over TCMF structure.

The TCMF biosensor was obtained through two steps: surface modification and bio-functionalization; the experimental processes are shown in Figure 5. First, since the sensor is calibrated to obtain RI sensitivity before surface modification and bio-functionalization, the waist area of TCMF was immersed in 5% HNO_3_ at room temperature for two hours before hydroxylation to fully clean the experimental reagent residues and other impurities on the surface of sensor. Second, the cleaned sensing area was immersed in the NaOH solution at room temperature for one hour, which formed the surface of the fiber with hydroxyl groups. The concentration of NaOH solution was 1 mol/L, which was prepared from deionized water. Third, the waist area was immersed in 5% (*v*/*v*) MPTMS ethanol solution for silanization so that the surface of the fiber had sulfhydryl groups; this was convenient for subsequent combination with MoS_2_. Fourth, the sensing area was immersed in the MoS_2_ solution with a concentration of 2 mg/mL to fix MoS_2_ on the sensor surface. The defect sites formed by the absence of sulfur atoms on the MoS_2_ surface can react with sulfhydryl groups. This functionalization technique is defined as organic mercaptan “ligand conjugation”, where mercaptan ligands may coordinate with Mo-atoms at S-vacancies, yielding a “ligand coordination” functionalization [23,24]. Fifth, the sensing area coated with MoS_2_ was immersed in a *T. gondii* Ag solution with a concentration of 100 μg/mL to immobilize the antigen. Due to the excellent hydrophobicity of MoS_2_, *T. gondii* Ag was successfully immobilized on the MoS_2_ functionalization sensing platform through hydrophobic interaction [17,25]. There were still blank sites on the surface of the biosensor that were not combined with *T. gondii* Ag. In order to block the non-specific bond of other biomolecules with the sensor in subsequent experiments, the sensing area was finally soaked with skimmed milk powder sealing fluid (SMPSF) with a concentration of 5% to close the blank sites on the surface of biosensor. 

After each of the above experimental steps was completed, the transmission spectrum of the sensor in PBS solution was recorded for comparative analysis of the experimental results. Finally, the prepared TCMF biosensor was immersed in different concentrations of *T. gondii* MAb solutions for specific detection to explore the immune detection ability of the biosensor. 

### 2.5. Experimental Setup

**Figure 6:**
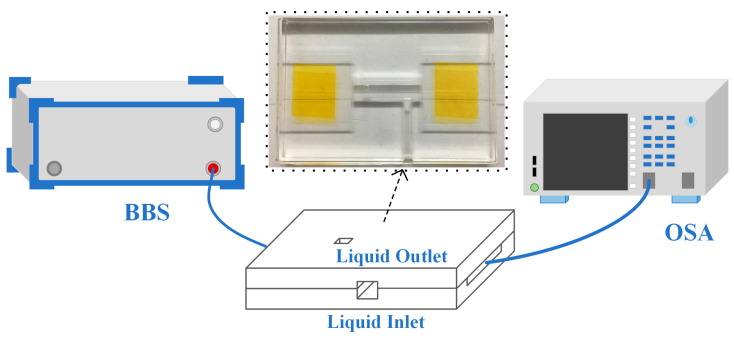
Experimental setup for immune sensing of TCMF biosensor.

Figure 6 shows the bio-sensing experimental setup, and the insert is a picture of the actual microfluidic chip. A broadband light source (BBS) with wavelength of 1250–1650 nm emitted a broadband light that was propagated through the input-SMF to the TCMF biosensor encapsulated by the microfluidic chip. Then, the optical signal was imported into the optical spectrum analyzer (OSA) through the output-SMF to obtain the spectral data. The proposed biosensor was injected into various sample solutions. The *T. gondii* MAb will specifically combine with the *T. gondii* Ag recognition unit on the surface, resulting in the change of RI near the sensing area. 

## 3. Results and Discussion

### 3.1. Characterization and Analysis of MoS_2_-Immobilized Structure

**Figure 7:**
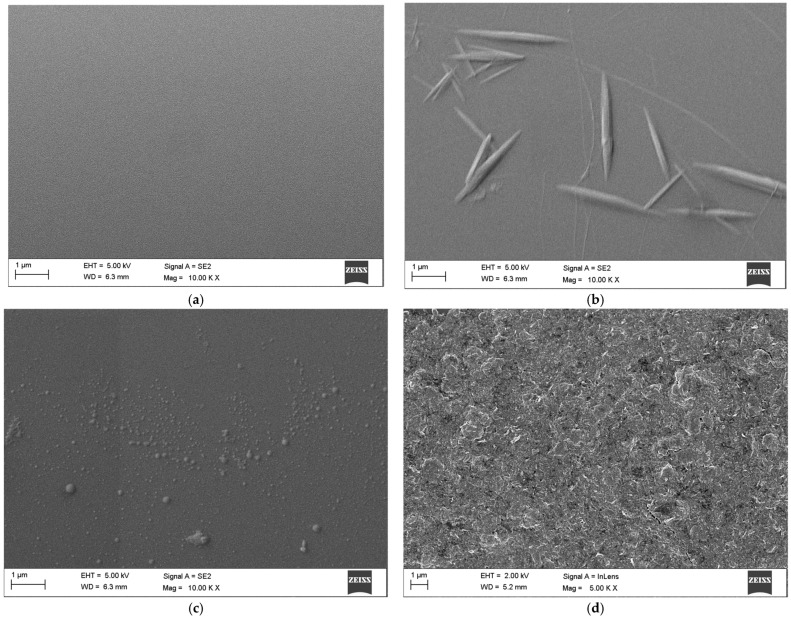
FESEM images of (**a**) bare TCMF sensor (**b**) TCMF sensor after hydroxylation (**c**) TCMF sensor after silanization (**d**) TCMF sensor coated with MoS_2_.

**Figure 8:**
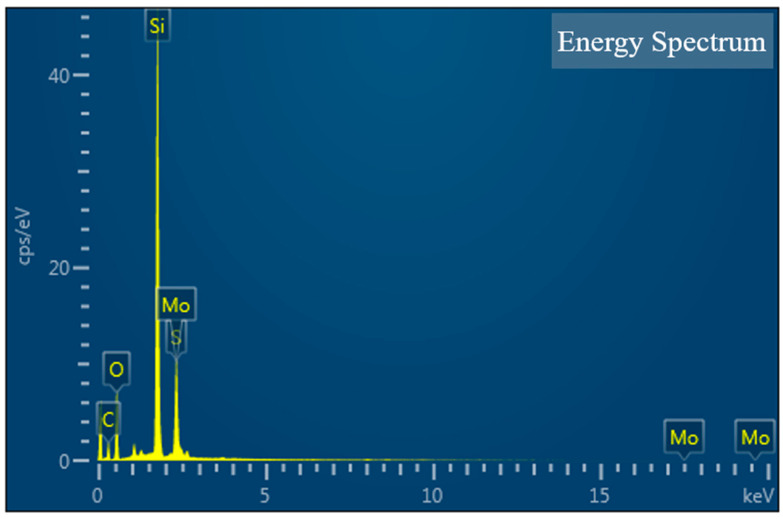
EDS image of MoS_2_ immobilized sensor structure.

The surface morphology of the TCMF sensor after surface modification was characterized by field emission scanning electron microscopy (FESEM). Figure 7a shows the morphology image of the bare fiber without surface modification, while Figure 7b,c show the surface morphology of the sensor after hydroxylation and silanization, respectively. The morphological characterization of the TCMF sensor modified with MoS_2_ is shown in Figure 7d. It can be clearly seen from Figure 7d that MoS_2_ was evenly coated on the surface of TCMF, and the uniform and dense MoS_2_ was conducive to binding with *T. gondii* Ag. In order to further characterize the coating performance of MoS_2_ on the sensor surface, the energy dispersive spectrometer (EDS) element composition was analyzed, as shown in Figure 8 and Table 1. Si and O are the main components of optical fiber materials; their mass fractions are 30.71% and 27.85%, respectively. C is the main component of chemical reagent used in the process of surface modification (such as MPTMS), and its mass fraction is 24.77%. The mass fractions of S and Mo are 7.06% and 9.61% and the atomic percentages are 4.22% and 1.92%, respectively. The atomic ratio is very close to the stoichiometric MoS_2_, which further indicates that MoS_2_ has been successfully modified on the sensor surface [23,25]. 

**Figure 9:**
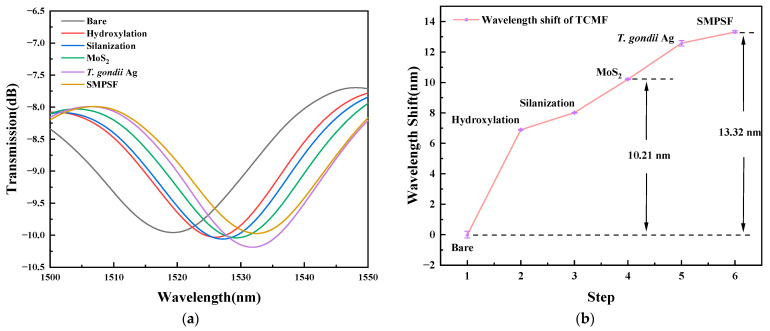
(**a**) Spectral response and (**b**) the corresponding wavelength shift of surface modification for TCMF biosensor placed in PBS solution.

Figure 9a,b show the spectral response and the wavelength shift of the TCMF sensor placed in a PBS solution after each modification step. It can be seen that the spectrum was redshifted during the modification process. This is because the effective RI of the cladding increased with the increase of the modification material on the sensor surface, resulting in the change of the effective RI difference between fundamental mode and high-order cladding mode [21]. In addition, the value of the red shift of the sensor coated with MoS_2_ and modified with *T. gondii* Ag is about 10.21 nm and 12.57 nm, respectively, indicating that the surface modification effect is good. It is worth noting that the sensor modified with *T. gondii* Ag biomolecules only has a red shift of about 0.747 nm during the sealing process, indicating that there are few blank binding sites on the sensor surface. Moreover, the standard deviation (SD) was about 0.0939, and the spectral data were recorded five times at each modification step.

### 3.2. Measurement of T. gondii MAb

**Figure 10:**
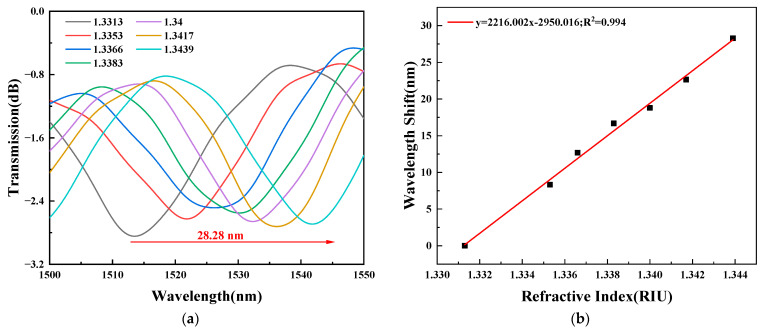
(**a**) Spectral response of the TCMF sensor. (**b**) Relationship between wavelength shift and ambient RI.

RI calibration was carried out to explore the sensitivity of TCMF biosensor before the biosensing experiments. According to the experimental device shown in Figure 6, the same volume of deionized water (RI was 1.3313) and sodium chloride solution with a concentration of 1% to 6% (corresponding RIs were 1.3353, 1.3366, 1.3383, 1.34, 1.3417 and 1.3439) were injected into the microfluidic chip, respectively. Figure 10a,b show the spectral response of the wavelength changing with the external environment RI and the linear fitting of the wavelength shift with the external environment RI, respectively. With the increase in RI, the wavelength of the TCMF sensor was redshifted; the value of red shift was 28.28 nm and RI sensitivity was 2216.002 nm/RIU. The relative standard deviation (RSD) was about 0.61%, and the spectral data were recorded five times. 

**Figure 11:**
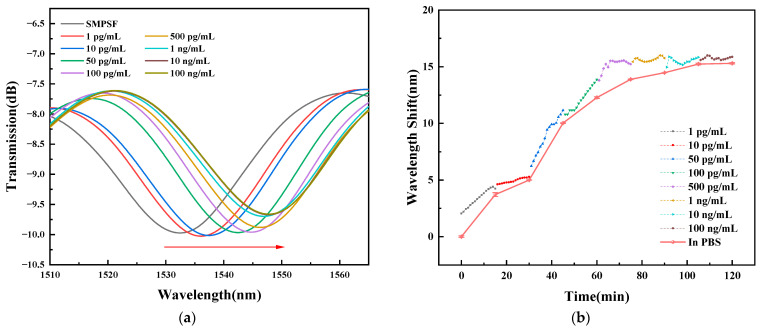
(**a**) Spectral evolution of the sensor to concentration of *T. gondii* MAb solutions, and (**b**) the corresponding wavelength shift with time.

Once it had finished surface modification and bio-functionalization, the TCMF biosensor was immersed in the *T. gondii* MAb solutions with a concentration of 1 pg/mL, 10 pg/mL, 50 pg/mL, 100 pg/mL, 500 pg/mL, 1 ng/mL, 10 ng/mL, and 100 ng/mL for immune detection. The volume of *T. gondii* MAb solution used in the experiment is 120 µL, smaller than 500 µL in Ref. [26] and 10 mL in Ref. [27], which has the characteristic of microscale detection. The reaction time of each concentration was fifteen minutes, and the spectral data were recorded every minute for fifteen minutes to obtain the dynamic reaction of *T. gondii* Ag and *T. gondii* MAb binding. After recording fifteen groups, five groups of spectral data in blank PBS solution were recorded repeatedly before immersion in the next concentration. The purpose of recording five groups of data was to obtain the average value to reduce experimental errors and avoid occasionality. The detection of *T. gondii* MAb solutions with different concentrations was completed in turn. The spectral data recorded by the biosensor in PBS solution after sealing was regarded as the initial wavelength reference point. The spectral response and wavelength shift with time in *T. gondii* MAb solutions of different concentrations are shown in Figure 11; the SD was about 0.0556. In addition, as can be seen in Figure 11b, eight groups of dotted lines with different colors represent the dynamic reaction process within fifteen minutes, while the solid line represents the mean value of wavelength shift in the five groups of PBS solutions. The figures show that the wavelength redshifted and the total value is 15.31 nm with the increase in concentration of *T. gondii* MAb solutions. That is because the combination of *T. gondii* MAb and *T. gondii* Ag caused the effective RI of different modes to change. Moreover, the wavelength shift is obvious in the concentration range of 1 pg/mL to 10 ng/mL, which is about 1.28 nm, 5.03 nm, 2.25 nm, 1.61 nm, 0.57 nm, and 0.76 nm, respectively, indicating intense binding. However, with the increase of concentration of *T. gondii* MAb solutions, the change of wavelength redshift tends to be gentle. This is because the *T. gondii* Ag sites on the TCMF biosensor surface that can specifically bind with *T. gondii* MAb gradually decrease. When the concentration increases from 10 ng/mL to 100 ng/mL, the wavelength redshift is about 0.067 nm with a slight redshift, indicating that the saturation point of the TCMF biosensor is about 10 ng/mL. Therefore, the detection range of the biosensor is about 1 pg/mL to 10 ng/mL. 

**Figure 12:**
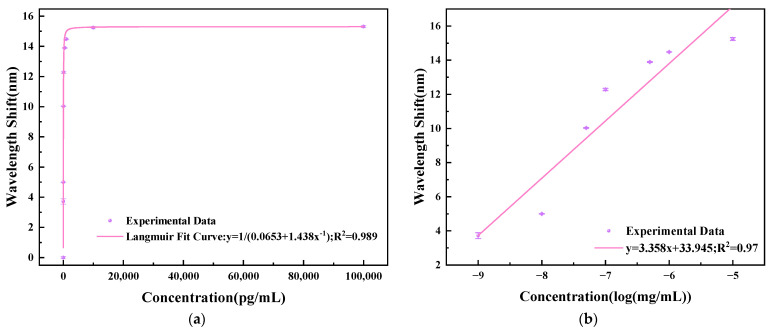
(**a**) Langmuir curve fitting of wavelength shift and *T. gondii* MAb concentration. (**b**) Relationship between wavelength shift and logarithmic concentration of *T. gondii* MAb.

A Langmuir curve was used to fit the relationship between wavelength shift in PBS solution and *T. gondii* MAb concentration after detection of each concentration, as shown in Figure 12a. The specificity adsorption of *T. gondii* MAb followed the Langmuir model, given by [28,29,30]:

(6) $$\frac{C}{\Delta \lambda }=\frac{C}{\Delta \lambda_{\max}}+\frac{K_{D}}{\Delta \lambda_{\max}}$$

where *C* is the concentration of *T. gondii* MAb solutions, Δλ is the resonant wavelength shift corresponding to the concentration, $\Delta \lambda_{\max}$ is the maximum resonance wavelength shift during detection, and $K_{D}$ is the dissociation constant of antibody-antigen reactions in the biosensor. As can be seen from Figure 12a, the Langmuir fitting curve is expressed as

(7) $$y=\frac{1}{0.0653+1.438x^{-1}}$$

with a good linear fit of 0.989. The dissociation constant *K_D_* and affinity constant *K_A_* of the TCMF biosensor can be calculated to be about 5.79 × 10^−13^ M and 1.727 × 10^14^ M^−1^, respectively. The dissociation constant is the equilibrium constant of the dissociation of antibody-antigen complex in the binding reaction between the antibody and site. The lower the dissociation constant is, the closer the binding is, which is inversely proportional to the affinity constant. The affinity constant indicates the compactness of antibody-antigen binding. The higher the value, the more tightly the antibody and antigen bind. The calculated results show that the biosensor has excellent affinity.

The calculation of the limit of detection (LOD) is based on the use of the calibration curve of the biosensor and on the International Union of Pure and Applied Chemistry (IUPAC) recommendation, which can be expressed as [31]

(8) $$x_{LOD}=f^{-1}({\bar{y}}_{blank}+3\sigma_{\max})$$

where ${\bar{y}}_{blank}$ is the mean value of the blank measurement, $\sigma_{\max}$ is the standard deviation of the blank measurement, and $f^{-1}$ is the inverse function of the Langmuir curve fitted by the experimental results. The LOD of the proposed biosensor is about 87 fg/mL, calculated from Equations (7) and (8), indicating the minimum value of analyte concentration that the sensor can detect. According to the methods in Ref. [32] and Ref. [33], the limit of quantitation (LOQ) is calculated to be about 287 fg/mL. It is concluded that the biosensor has extremely low LOD and can be used to detect biomolecules with lower concentration. It is expected to detect trace analytes quickly and accurately and realize biomedical applications. 

Within the detection range of 1 pg/mL to 10 ng/mL, the relationship between the wavelength shifts of the TCMF biosensor and the logarithm concentration of *T. gondii* MAb solution was obtained, as shown in Figure 12b. It can be seen from the figure that the linear fitting curve is expressed as

(9) y=3.358x+33.945

It was concluded that an ultra-high detection sensitivity of 3.358 nm/log(mg/mL) with a linear fit of 0.97 was obtained; the SD was about 0.0528. 

In order to visually demonstrate the sensing performance of the proposed biosensor, Table 2 shows a comparison of the performance of the biosensor prepared in this paper with other reported sensing platforms. It can be seen from the table that the proposed biosensor has a lower LOD and higher detection sensitivity, which proves the significant advantages of the proposed biosensor. 

**Figure 13:**
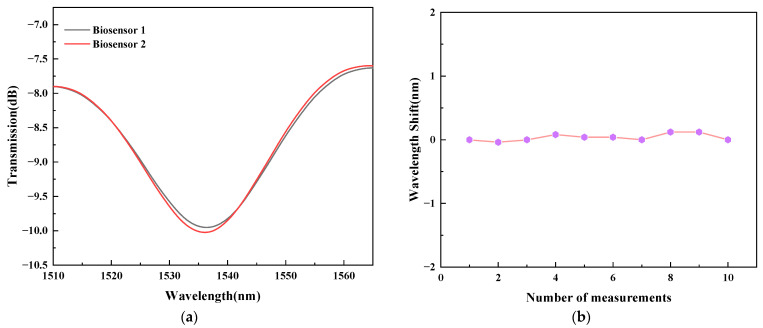
(**a**) Reproductivity, and (**b**) stability of the biosensor.

Reproductivity and stability are important indicators for evaluating the sensing performance of sensors. Two TCMF biosensors with similar parameters were prepared to detect *T. gondii* MAb solution with a concentration of 1 pg/mL to evaluate reproductivity. Figure 13a shows the transmission spectra of two biosensors. It can be seen from the figure that the spectral changes of two biosensors are not significant, indicating that the proposed sensor has good reproductivity. Moreover, to evaluate the stability of the sensor, ten groups of data were recorded from the sensor in a blank PBS solution to determine the impact of multiple tests on sensing performance. Figure 13b shows the variation of the wavelength shifts of the sensor with the number of measurements. It can be seen from the figure that the wavelength change of the sensor is very small. In addition, its standard deviation is 0.05481. From the above experimental results, it can be concluded that the sensor has good stability and accuracy. 

### 3.3. Specificity and Clinical Test

**Figure 14:**
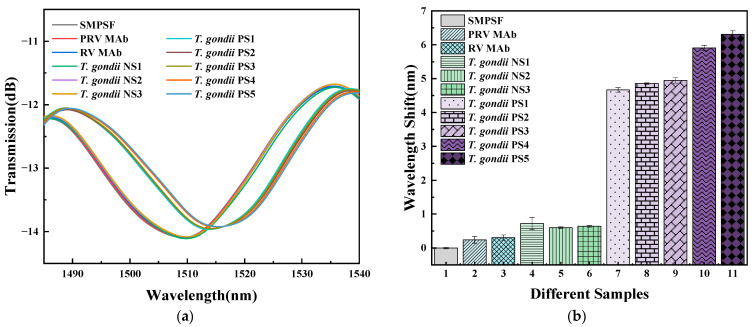
Specificity and clinical tests of the TCMF biosensor for (**a**) spectral response and (**b**) wavelength shift.

In order to characterize the specificity and clinical immunity in complex biological environments, the biosensor was immersed in 5% HNO_3_ for three hours to remove the coating on the sensor surface. Then, surface modification and bio-functionalization were performed again according to Section 2.4 to obtain the modified biosensor. The specificity and clinical performance of the TCMF biosensor were tested using the pseudorabies virus monoclonal antibody (PRV MAb) with a concentration of 1 ng/mL, rabies virus monoclonal antibody (RV MAb) with a concentration of 1 ng/mL, and clinical samples of *T. gondii* negative serum and *T. gondii* positive serum. The reaction time of each solution was also fifteen minutes. Before detecting the next solution, the surface of the biosensor was cleaned five times with PBS solution, and then five groups of spectral data in blank PBS solution were recorded repeatedly. The specificity and clinical tests were completed in turn, and the spectral data recorded by the biosensor in PBS solution after sealing was used as the reference point of the initial wavelength. The detection results are shown in Figure 14; the SD was about 0.0263. It can be seen that the interference spectrum has a slight redshift for PRV MAb and RV Mab; the redshift values are 0.24 nm and 0.31 nm, respectively. The biosensor has no binding ability to PRV MAb and RV MAb, as there are no *T. gondii* MAbs in the two solutions. The experimental results demonstrate that the biosensor has specificity binding to *T. gondii* MAb.

Three groups of clinical samples of *T. gondii* negative serum (labeled as *T. gondii* NS1, *T. gondii* NS2, and *T. gondii* NS3) and five groups of clinical samples of *T. gondii* positive serum (labeled as *T. gondii* PS1, *T. gondii* PS2, *T. gondii* PS3, *T. gondii* PS4, and *T. gondii* PS5) were diluted at a ratio of 1:20 to explore clinical performance. As can be seen from Figure 14, the proposed biosensor has only slight redshift in clinical samples of *T. gondii* negative serum, with redshift amounts of 0.72 nm, 0.6 nm, and 0.64 nm, respectively; it has significant redshift in clinical samples of *T. gondii* positive serum, with redshift amounts of 4.67 nm, 4.85 nm, 4.95 nm, 5.91 nm, and 6.31 nm, respectively. The reason for the red shift of the interference spectrum is that the clinical samples of *T. gondii* positive serum contain *T. gondii* MAb that can combine with the *T. gondii* Ag on the biosensor surface. The wavelength shift of the proposed biosensor in the clinical samples of positive serum is different from that in *T. gondii* MAb solutions in Section 3.2. The reason is that the composition of the serum is very complex. In addition to *T. gondii* MAb, it also contains essential nutrients, various growth factors, hormones, and various proteins and enzymes, while the *T. gondii* MAb solutions used in Section 3.2 is the antibody protein of high purification. Based on the advantages of excellent specificity and clinical performance, the proposed biosensor has great potential in biosensing fields. In addition, the sensor can be reused after the coating is removed by HNO_3_, indicating that the proposed sensor has excellent repeatability and recovery. 

## 4. Conclusions

A microfluidic biosensor with TCMF was proposed and demonstrated in this study. The sensor had been fabricated using splicing of a section of TCF with two sections of SMF, thereafter TCMF was obtained by arc discharging and flame heating. Moreover, the present study provided significant improvement in surface modification and bio-functionalization of optical fiber biosensor, particularly the biosensor for rapid analysis of *T. gondii* monoclonal antibodies. MoS_2_, with the advantages of large surface-to-volume ratio and the existence of free sulfur groups in the hydrophobic interaction, was coated on the surface of the TCMF sensor, and the morphology of TCMF sensor after surface modification was characterized by FESEM to verify the effectiveness of the scheme. The MoS_2_ was interfaced with TCMF sensor through the “ligand coordination” functionalization and then effectively bio-conjugated with *T. gondii* antigens via hydrophobic interaction. Then, the label free detection of *T. gondii* MAb was achieved with high precision by observing the wavelength shifts. The experimental results indicated that the detection range of the proposed biosensor was 1 pg/mL to 10 ng/mL, the LOD was calculated to be 87 fg/mL, with sensitivity of 3.358 nm/log(mg/mL), and the dissociation constant and affinity constant of 5.79 × 10^−13^ M and 1.727 × 10^14^ M^−1^, respectively. In addition, the detection results among RV MAb, PRV MAb, *T. gondii* positive serum, and *T. gondii* negative serum confirmed that the proposed biosensor showed excellent specificity and clinical performance for *T. gondii* Mab, even in complex clinical serum environment. In summary, the proposed microfluidic biosensor has the advantages of simple structure, easy construction, and excellent specificity and clinical performances, assuring its promising application prospects in biosensing fields. 

## Figures and Tables

**Table 1:** Element composition of MoS_2_ immobilized sensor structure.

Chemical Elements	Mass Fraction	Atomic Percentage
C	24.77	39.54
O	27.85	33.36
Si	30.71	20.96
S	7.06	4.22
Mo	9.61	1.92
Amount	100.00	100.00

**Table 2:** Comparison of the proposed study with the reported sensing platforms.

Sensor Type	Modification Material	Analyte	LOD	Detection Sensitivity	Ref.
U-shape fiber	MoS_2_/Au	Human IgG	19.7 ng/mL	1.014 nm/(µg/mL)	[34]
Plastic clad silica fiber	MoS_2_/SnO_2_	Creatinine	1.86 µg/mL	0.41 nm/(µg/mL)	[35]
Etched multimode fiber	Au/MoS_2_	BSA	0.29 µg/mL	0.9234 nm/(µg/mL)	[17]
Etched MPM fiber structure	GO/AuNPs/MoS_2_-NPs	cTnI	96.2638 ng/mL	3.4 pm/(ng/mL)	[26]
TCMF	MoS_2_	*T. gondii*	87 fg/mL	3.358 nm/log(mg/mL)	This work

## Data Availability

Not applicable.
